# A new method for estimating recent adult mortality from summary sibling histories

**DOI:** 10.1186/s12963-024-00350-0

**Published:** 2024-11-12

**Authors:** Bruno Masquelier, Ashira Menashe-Oren, Georges Reniers, Ian M. Timæus

**Affiliations:** 1 Center for Demographic Research, University of Louvain (UCLouvain), Louvain-la-Neuve, Belgium; 2 Department of Population Health, London School of Hygiene and Tropical Medicine, London, UK; 3 Centre for Actuarial Research, University of Cape Town, Cape Town, South Africa

**Keywords:** Adult mortality, Indirect estimation, Sibling survival histories, Demographic and Health Surveys

## Abstract

**Background:**

In low- and middle-income countries with limited death registration statistics, adult mortality rates are commonly estimated through sibling survival histories (SSH). In full SSH, respondents are asked about either the age, or the age and time of death, of each of their siblings in turn. Full SSH allow direct mortality estimation but can be time-consuming to collect. In this study, we introduce a new indirect estimation method using summary SSH, requiring only a limited set of questions to produce recent mortality estimates.

**Methods:**

We developed a set of 192 microsimulations representing a wide range of fertility and mortality patterns, and reconstructed summary SSH within these simulations as if they had been collected from adults aged 15–49. For each age group of respondents, we calculated coefficients that convert the proportion of adult siblings who died in the previous 5 years into age-specific mortality rates. We then evaluated the performance of this new method with real data, using 154 Demographic and Health Surveys.

**Results:**

The new indirect method provides mortality rates that are consistent with direct estimates from full SSH. Across all DHS, the mean absolute percentage error in the risk of dying in adulthood (ages 15–49) is 6% for both men and women. In all but one survey, 95% confidence intervals around the direct and indirect estimates overlap. As with direct estimates of adult mortality from SSH, the indirect estimates remain, however, lower than those of the Population Division of the United Nations.

**Conclusions:**

Summary questions on sibling survival can be included in censuses and rapid turn-around surveys for the measurement of recent adult mortality.

**Supplementary Information:**

The online version contains supplementary material available at 10.1186/s12963-024-00350-0.

## Introduction

In many low- and middle-income countries, systems of civil registration and vital statistics are underdeveloped, and improvements in the completeness of death registration have been slow [1]. Population-based surveys and censuses thus remain key data sources for estimating mortality, especially among adults [2, 3]. Since the 1990s, the sibling survival histories (SSH) collected by several programmes, most notably the Demographic and Health Surveys (DHS), have helped to fill the gaps that exist in the data on mortality between ages 15 and 59.

In full SSH, adults aged 15–49 are asked about all maternal siblings, and provide details on their gender, and age at the survey if still alive. For the deceased siblings, information is collected on age at death and years since death. All-cause mortality rates are obtained by dividing the number of deaths in each desired period and age group by the total number of years of exposure. Additional questions enable one to identify cause-specific mortality such as pregnancy-related or violent deaths. Full SSH have been used to monitor mortality increases due to HIV/AIDS [4, 5], to measure the effectiveness of health programmes [6], or, to study excess mortality associated with violence [7–9]. Full SSH are also one of the primary sources of data on adult survival used by the Global burden of disease (GBD) study [10] and the World population prospects (WPP) [11].

Although SSH are widely utilized, they have certain limitations. For example, they cannot be used to estimate mortality on a sub-national scale, and the uncertainty surrounding estimates is such that it is often necessary to pool different surveys or resort to modelling to reconstruct mortality trends [4]. SSH may also be subject to errors. There are relatively few missing responses on gender, survival status, or age at the time of the survey in the SSH collected in DHS [12]. Respondents seem to find it more challenging to place deaths in time, however, resulting in a higher proportion of missing responses on the timing of deaths as well as heaping on the time since death (especially 10 years prior to the survey). Further, deaths that occurred more than 8–10 years before the survey are underreported more often than recent deaths [5, 13]. Finally, because respondents are part of the group about which they provide information, and some sibships may appear multiple times in the sample, selection bias might be introduced [14, 15].

Another important caveat concerning full SSH is that their collection can be time-consuming. Consequently, about half of Demographic and Health Surveys omit the sibling module, and census questionnaires never incorporated full SSH. Recent mobile phone surveys to measure mortality also chose not to use full SSH, to avoid respondent fatigue and limit potential network interruptions^1^. Summary SSH can address this caveat. Compared to full SSH, collecting summary SSH can reduce interview duration by approximately 10 min, roughly cutting the interview time for SSH in half [16]. The framing of questions for summary SSH can vary, but respondents are typically asked about the total number of sisters/brothers ever born, and the number of these sisters/brothers who have died. Mortality rates are indirectly derived from the proportions of surviving siblings, tabulated by the respondent’s age.

Indirect estimation is possible with data from DHS (using the summary data collected before the full SSH questions) and a selection of multiple indicator cluster surveys (MICS) and CDC’s Reproductive Health Surveys. Some censuses have also collected summary SSH (e.g. Dominican Republic in 2002, Eswatini in 2007, and Lesotho in 2006). When first introduced, summary SSH were only asked of sisters and associated with an additional question regarding the number of sisters who died during pregnancy, childbirth, or within six weeks after the end of pregnancy. The proportions of adult sisters who died from pregnancy-related causes can be converted into pregnancy-related mortality rates using the “sisterhood method” developed by Graham et al. [17]. Timæus et al. in 2001 extended this method to all-cause mortality, including the estimation of men’s mortality from data on brothers [18]. To apply their method, respondents should have been asked about the number of their siblings who survived to the age of 15 and how many of them are still alive. No additional information is required regarding the ages or timing of the death of siblings.

The indirect method has three significant limitations, all related to the timing of deaths. First, estimates cover long reference periods, with data from respondents aged 20–24 reflecting mortality experiences three years before data collection, and data from those aged 45–49 yielding estimates for a period up to 15 years prior to the survey. Second, the method assumes regular and unidirectional mortality trends, making it unsuitable for settings disrupted by conflict, disasters, or epidemics that may cause sudden increases in mortality. Third, there is an increased likelihood of omissions in respondents’ reports because the calculation includes sibling deaths from the distant past [5].

To address these limitations we introduce a novel approach, focusing exclusively on recent deaths and building on the literature on synthetic cohorts for mortality estimation [19, 20]. The method requires three questions for each sex: the number of siblings who have reached age 15, the number who have died, and the number of deaths in the last five years. These questions make it possible to obtain the proportions of siblings who have died in the last five years, among all those who have reached adulthood, for each age group of respondents. These proportions can be converted into conditional probabilities of dying referring to the recent past.

In introducing this new method, we use a set of microsimulations to generate coefficients to convert reports on sibling deaths to mortality estimates. We show in detail how this method differs from the direct estimation method, and the original indirect method of Timæus et al. [18]. We evaluate the performance of both indirect approaches, comparing estimates obtained from 154 DHS, and in comparison to the WPP.

## Existing indirect methods for summary sibling histories

Using theoretical models of stable populations, the proportion of surviving siblings can be expressed as a function of age patterns of mortality and fertility, and the population growth rate. For example, if we denote the current age of a respondent as *a*, the mother’s age at the time of the respondent’s birth as *x* and the age interval between the respondent and their siblings as $x - y$, the number of surviving siblings born before the respondent is obtained as follows [21]:

1 $$\begin{aligned} S^{old, surv}(a) = \int ^{\beta }_{\alpha } \int ^{x}_{\alpha } m(y) e^{-rx}\,_{x}p_{0}\,m(x) \, _{a+x-y}p_{0} dy dx \end{aligned}$$

where *m*(*x*) refers to the fertility schedule, $_{x}p_{0}$ to the life table survivorship to age *x* and *r* to the growth rate in a stable population. A similar equation exists for surviving siblings born after the respondent. Hill and Trussell [22] used these expressions to convert the proportions of surviving siblings into life table survivorship, accounting for all deaths, including those that occurred in childhood. Through simulations of various stable populations, they generated 324 sets of proportions of surviving siblings by age group of the respondent ($_{5}S_{n}$). These proportions were related to the underlying probabilities of survival using the following equation:

2 $$\begin{aligned} _{n}p_{0} = \beta _{0}(n) + \beta _{1}(n)_{5}S_{n} \end{aligned}$$

Despite its potential, this indirect method has not been widely adopted, because the proportions are prone to errors as the respondent may omit siblings, especially those who died before the respondent reached maturity. A comparison of sibship sizes reported in SSH with sibship sizes expected from fertility trends suggested that around 15% of siblings were unreported in DHS, mostly due to omissions of siblings who died in childhood and/or during the childhood of the respondent [23].

What one assumes about the age differences between siblings is important. Graham et al. [17] assumed that the distribution of age differences between the respondent and her sisters remains constant across different ages of respondents, is normally distributed, and is centred around zero when the sibships are complete, i.e. when the mothers of the respondents have concluded their reproductive period. This assumption amounts to considering that the age of the respondents is our best approximation for the age of their sisters. However, Garenne and Friedberg [24] evaluated the performance of the sisterhood method through simulations and identified several issues: indirect estimates were consistently higher than direct estimates, and had a large margin of error. The pregnancy-related mortality rates inferred from the youngest respondents appeared particularly biased. One key source of this bias can be traced back to the assumption about age differences.

Timæus et al. [18] showed that the average difference between the age of an individual and the age of their siblings is zero only in stationary populations. In populations with a positive growth rate, this difference is negative, as respondents tend to have more younger siblings than older ones. Conversely, in populations with a negative growth rate, the difference is positive. In addition, Timæus et al. [18] highlighted that these distributions have varying standard deviations, whereas Graham et al. assumed that it was fixed [17]. Theoretically, in a cohort of women who have completed childbearing, the variance of the distribution of age differences between siblings should be approximately twice the variance of the distribution of intervals between the first birth and all subsequent births— referred to by Timæus and colleagues as the “birth distribution” [18]. However, this birth distribution is not easily observed, and its variance cannot be inferred from knowledge of the fertility schedule measured at the aggregate level. If there is a lot of variance around ages at first birth, the variance of the birth distribution will be smaller than the variance of the fertility schedule. Timæus et al. [18] used data from 12 World Fertility Surveys and confirmed that the variance of the birth distributions was smaller than twice that of the fertility schedule.

Timæus et al. [18] then developed an indirect method for estimating all-cause mortality from proportions of siblings who survived to the time of data collection, among those who reached their 15th birthday ($_{5}S^{15+}_{n}$). In contrast to the method proposed by Hill and Trussell (1977) [22], they excluded siblings who died in childhood from the numerator and denominator. We will refer to these proportions as “adult lifetime proportions” because the death of adult siblings might have happened at any point in time in the past. Using stable population theory, they expressed $_{5}S^{15+}_{n}$ as a function of mortality and fertility rates. They generated 192 different stable populations, and related the proportions of surviving siblings to survivorship probabilities with linear regression:

3 $$\begin{aligned} _{n-15}p_{15} = \beta _{0}(n) + \beta _{1}(n)\times _{5}S^{15+}_{n-5} \end{aligned}$$

The $\beta _{0}$ and $\beta _{1}$ coefficients are provided in the appendix (Table A1). They tend to cancel each other out, suggesting that the adjustments to convert the proportions $_{5}S^{15+}_{n-5}$ into survival probabilities $_{n-15}p_{15}$ are small. The method should therefore not be very sensitive to variations in fertility and mortality age schedules. Reports provided by those under age 20 are discarded, as siblings are on average older than the respondents aged 15–19, rendering the estimates derived from this age group quite sensitive to the choices made in modelling the distribution of age differences between a respondent and his or her siblings. Estimates obtained from older respondents (e.g. 45–49) refer to an earlier period than those provided by younger respondents (e.g. 20–24). In order to date the estimates, Timæus et al. [18] computed coefficients to estimate the time elapsed between the reference period and the survey, assuming a smooth and unidirectional change in mortality.

## Data and methods

### Sibling histories from Demographic and Health Surveys

We use 154 Demographic and Health Surveys that included a module on sibling mortality (Online Appendix, Table A2). These surveys were conducted between 1992 and 2022 in 54 countries. SSH follow a similar structure to the full birth histories collected to measure fertility and child mortality but focus on the children of the respondent’s mother.

Direct and indirect calculations typically leave out the respondent, as the respondent is by definition a survivor. This does not introduce bias into the estimates, as long as the number of adult siblings alive in recent years is not associated with mortality. This is because the exclusion of the respondent in SSH is offset by the absence of data on sibships without survivors and the greater likelihood that a low-mortality sibship will be found multiple times in the sample [15, 25]. Other approaches to dealing with selection biases have been proposed [14, 26].

### Direct estimation based on full sibling histories from a single survey

Direct mortality estimation from full SSH consists of counting deaths and exposure time for a given reference period, by sex and age group [27]. Discrete-time regression models can be used to model trends or patterns by age [4]. Several R packages are available to facilitate direct estimation (e.g. demogsurv,^2^DHS.rates^3^ and siblingsurvival^4^).

An example of direct calculation is provided in Table 1 for the 2015 DHS conducted in Zimbabwe. Estimates are presented for two reference periods: (1) 0–6 years before data collection, consistent with the published DHS reports, and (2) 0–4 years prior to data collection, which aligns with the duration for computing estimates using the new indirect method described below. The calculation is based on months of birth and death imputed by the DHS program from responses given to questions about age at the time of survey, or age at death and time since the death. The age-specific mortality rates ($_{n}m_{x}$) are converted in risks of dying ($_{n}q_{x}$) and chained together to obtain the summary index $_{35}q_{15}$. Confidence intervals around the age-specific mortality rates and the probability $_{35}q_{15}$ are obtained using a stratified jackknife approach. The female $_{35}q_{15}$ probability for 0–6 completed years before the survey is 281.6‰ (95% CI: 254.5$-$307.7), which is consistent with the published report (282‰) [28]. The probability $_{35}q_{15}$ calculated for 0–4 years before the survey is slightly lower, estimated at 245.4‰ (95% CI: 216.8$-$272.9).

**Table 1:** Direct calculation of age-specific mortality rates from the 2015 Zimbabwe DHS, female mortality, 0–6 and 0–4 years prior to the survey

n	Age group	Nb. of deaths	Person-years	ASMR (‰)	$_{5}q_{n}$ (‰)	95% CI (‰)
0-6 years before data collection						
15	15–19	30	15812	1.9	9.4	5.0–13.7
20	20–24	49	19840	2.5	12.3	8.6–16.2
25	25–29	113	22540	5.0	24.8	19.6–30.0
30	30–34	212	19692	10.8	52.4	44.2–60.5
35	35–39	180	13168	13.7	66.1	55.3–76.5
40	40–44	123	8046	15.3	73.6	59.6–86.9
45	45–49	83	4831	17.2	82.4	60.4–103.1
				$_{35}q_{15}$ = 281.6‰ (95% CI: 254.5–307.7)		
0-4 years before data collection						
15	15–19	23	10590	2.2	10.8	5.1–16.0
20	20–24	32	13645	2.3	11.7	7.2–16.4
25	25–29	72	15908	4.5	22.4	16.1–28.5
30	30–34	141	14942	9.4	46.1	36.9–55.2
35	35–39	108	10054	10.7	52.3	39.9–64.1
40	40–44	85	6270	13.6	65.5	49.9–80.8
45	45–49	50	3699	13.5	65.4	43.8–87.3
				$_{35}q_{15}$ = 245.4‰ (95% CI: 216.8–272.9)		

### Indirect estimation based on adult lifetime proportions computed from a single survey

The indirect method proposed by Timæus et al. [18] can be applied to DHS data, using only the information on the mean number of siblings born to the same mother who have reached 15 years of age and the number of these siblings who are still alive at the time of the survey. The calculation is illustrated in Table 2, again based on the 2015 Zimbabwe DHS. We used the West model of Princeton life tables to convert survivorship ratios into the summary index $_{35}q_{15}$ [29]. The average of values obtained from 20–29-year-old respondents (269.1 ‰) is only about 4% lower than the direct estimate referring to the 0–6 year period before the survey (281.6‰) and is contained in the corresponding 95% confidence interval (254.5$-$307.7‰). Indirect estimates suggest, however, that mortality remained fairly stable over time. For Zimbabwe, this is not plausible as mortality declined owing to the roll-out of antiretroviral therapy during this period [30]. This decline is not well reflected here, most likely due to the assumption of linearity of trends required to date the estimates. It is also possible that the conversion of the age-specific estimates of $_{n-15}q_{15}$ into the summary index $_{35}q_{15}$ using a standard mortality pattern introduced biases since this pattern does not capture excess adult mortality in populations with generalized HIV epidemics.

**Table 2:** Indirect calculation of age-specific mortality rates from the 2015 Zimbabwe DHS, female mortality, 0–14 years prior to the survey

n	Age group	Sisters reaching age 15	Sisters surviving	$_{5}S^{15+}_{n-5}$	$_{n-15}p_{15}$	Years since survey	$_{35}q_{15}$ (‰)	95% CI (‰)	
25	20–24	2580	2441	0.946	0.947	3.3	245.0	208.2–281.0	
30	25–29	3370	3055	0.906	0.893	5.7	293.3	259.5–326.3	
35	30–34	3787	3361	0.887	0.870	7.9	259.1	231.9–285.7	
40	35–39	3110	2648	0.851	0.832	10.0	256.0	231.0–280.7	
45	40–44	2578	2074	0.805	0.785	12.0	262.0	236.9–287.1	
50	45–49	1543	1145	0.742	0.719	13.9	281.1	250.3–312.0	

### Indirect estimation based on proportions of adult siblings who have died in the last five years, computed from a single survey

We now introduce the new indirect approach that uses an additional question to identify recent deaths. The method requires that the following three questions are asked:

1. How many of your sisters born to the same mother have reached age 15?
2. How many of these adult sisters have died?
3. How many of these adult deaths occurred during the last 5 years?

The same questions can be asked about brothers to estimate male mortality.

The first two questions enable one to compute $_{5}S^{15+}_{n} (t)$, the proportions of siblings who survived to the time of data collection (*t*), among those who reached their 15th birthday, to apply the method developed by Timæus et al. [18] and reconstruct past trends.

With the last question, it also is possible to compute $_{5}S^{15+}_{n-5}(t-5)$, the proportion of siblings who have reached age 15 that were alive 5 years previously ($t-5$), calculated from reports of respondents aged from $n-5$ to *n* years. The relative changes in this proportion as each cohort of respondents ages from the age group $n-5$ to *n* to the age group *n* to $n+5$ are indicative of the impact of adult mortality over the five years.

Theoretically, it should be possible to use the coefficients developed by Timæus et al. [18] in this context, after chaining the cohort changes in the proportions across age groups to refer to a synthetic cohort. This approach was first proposed by Zlotnik et al. (1981) [19] for indirect estimation based on child and parental survival, and could be extended to sibling histories. However, one would need to account for the fact that some siblings reach 15 years during the 5-year window period, and these siblings are less likely to die compared to those who already are 15 at time $t-5$. This complicates the construction of a synthetic cohort.

We, therefore, developed new coefficients that can be applied directly to the ratio $\frac{_{5}S^{15+}_{n}(t)}{_{5}S^{15+}_{n-5}(t-5)}$. Previous indirect sibling methods were developed using mathematical expressions that relate the proportions of surviving siblings to mortality patterns in stable populations. This approach allows some freedom in the generation of stable populations and in the choice of regression model specifications but lacks flexibility when using estimators that are difficult to express mathematically. Here, we still work with stable populations, but rather than exploiting their mathematical expression, we simulate them. Through microsimulations of stable populations, we can directly observe sibships and compute the required estimators [15].

We use SOCSIM, an open-source computer program designed to simulate population dynamics. Starting from a list of individuals with basic characteristics, it generates life events such as births, deaths, marriages and divorces [31–36]. The waiting time for each event is stochastically determined using a competitive risk model that considers predefined demographic rates. SOCSIM operates as a closed model, meaning individuals can only enter the simulation through birth and exit through death. This closed model structure facilitates the identification of sibships, ensuring that all individuals born during the simulation have an identified mother. Respondents, selected from adults aged 15–49 at the end of the simulation, have their sibling survival histories reconstructed by identifying all individuals born to the same mother.

To create the populations, we model mortality using relational logit models, specifying four values for the $\alpha _{m}$ parameter and two values for the $\beta _{m}$ parameter, based on Brass’s general standard [37] (Fig. S1). We model fertility using the Brass relational model, with four values of $\alpha _{f}$ capturing the age pattern of the fertility schedule, four values of $\beta _{f}$ describing the spread of the fertility schedule [38], and the standard developed by Booth (1984) for populations with high fertility [39]. The initial age structure of the population is calculated based on the survival curve, shape of the fertility schedule, and two values of the growth rate (0.01 or 0.03). The initial population size is set to reach approximately 40,000 surviving members after 150 years of simulation, allowing sufficient time for the population to grow and eliminate individuals with no pre-established kinship relationships at the start.

The main parameter values for the simulations are detailed in Table 3. These are identical to those used by Timæus et al. [18] to estimate the relationship between mortality and sibling survival. The resulting 192 simulations encompass a wide range of demographic profiles, with life expectancies at birth ranging from 35.4 to 74.1 years and the mean age of the fertility schedule ranging from 25.5 to 31.3 years.

In the Online appendix (A.3), we show that the microsimulation model is well calibrated. Mortality rates recalculated from the simulated populations for the last 5 years are consistent with the life tables introduced as input parameters, and direct estimates of the age-specific mortality rates from the reconstructed SSH provide unbiased estimates of the life table probability $_{35}q_{15}$, the risk that a person aged 15 dies before reaching age 50 (Fig. A3). We also apply the indirect method proposed by Timæus et al. [18] to the microsimulations and recover unbiased estimates of the probability $_{35}q_{15}$. This is expected since we constructed the simulations from the same stable populations as those used to develop the coefficients, but it also indicates that assumptions made by Timæus et al. [18] to approximate age differences between siblings are well captured by the simulations.

**Table 3:** Parameters used to set up the microsimulations

Mortality	Brass general standard
$\alpha _{m}$	− 1.0, − 0.6, − 0.2, 0.2
$\beta _{m}$	0.7, 1.1

Fertility	Booth standard
$\alpha _{f}$	− 0.5, − 0.2, 0.1, 0.4
$\beta _{f}$	1.0 (*r* = 0.03), 1.15, 1.4, 1.8 (*r* = 0.01)

Growth rate	
*r*	0.01, 0.03

Using the 192 populations, we used linear regression to predict life table survivorship from the aggregate reported proportions of adult siblings who were still alive at the time of the survey, among those who were alive 5 years before, as follows:

4 $$\begin{aligned} _{5}p_{n} = \beta _{0}(n) + \beta _{1}(n)\times \frac{_{5}S^{15+}_{n}(t)}{_{5}S^{15+}_{n-5}(t-5)} \end{aligned}$$

The $\beta _{0}$ and $\beta _{1}$ coefficients are provided in Table 4. Other specifications were explored, such as regressing on $_{5}p_{n-5}$, but this simple model represents greater variance and has the additional advantage of providing estimates for all age groups. When questions are asked among respondents aged 15–49, the resulting estimates of $_{5}p_{n}$ can be chained together to obtain the summary probability $_{35}q_{15}$ as $1 - \prod _{n=15}^{45}\ _{5}p_{n}$. Although the coefficients have been calculated in a universe of stable populations, in practical applications it is not necessary to invoke the stability assumption because mortality is measured over a short interval of five years before the survey.

**Table 4:** Coefficients used to convert proportions of adult siblings who are still alive at the time of the survey, among those who were alive 5 years before

*n*	Age group	$\beta _{0}$	$\beta _{1}$	$R^{2}$	CV
15	15–19	0.0535	0.9459	0.9200	0.0039
20	20–24	﻿− 0.2408	1.2404	0.9323	0.0049
25	25–29	﻿− 0.0752	1.0742	0.9320	0.0050
30	30–34	0.0169	0.9829	0.9116	0.0055
35	35–39	0.0151	0.9843	0.9002	0.0067
40	40–44	− 0.0273	1.0268	0.9061	0.0078
45	45–49	− 0.0332	1.0326	0.9126	0.0086

An example of the calculation is provided for the 2015 Zimbabwe DHS in Table 5. The proportions $_{5}S^{15+}_{n}(t)$ are reconstructed on the basis of the full SSH, assuming that the respondents would have provided the same information on the number of their siblings over 15 years old, the number who have died, and the number of deaths in the last five years if they had been asked only the summary questions. The $_{35}q_{15}$ probability obtained with this indirect method is 228.8‰ (95% CI: 205.2–252.5), which is only 7% lower than the direct estimate computed from full SSH (Table 1). These two estimates are not significantly different. There are, however, some notable deviations in the age-specific mortality rates. These are higher than the direct estimates when deduced from summary data provided by younger respondents, and lower when derived from older respondents, but the confidence intervals overlap for five of the seven age groups. A more systematic comparison is presented in the Results section, based on all available DHS.

**Table 5:** Indirect calculation of age-specific mortality rates using proportions of adult siblings who are still alive at the time of the survey, among those who were alive 5 years before, 2015 Zimbabwe DHS, female mortality

n	Age group	$_{5}S^{15+}_{n-5}$	$_{5}S^{15+}_{n}$	$_{5}p_{n}$	$_{5}q_{n}$	95% CI
		$(t-5)$	(*t*)	(‰)	(‰)	(‰)
15	15–19	0.982	0.963	981.1	18.9	10.0–27.8
20	20–24	0.968	0.946	971.4	28.6	20.6–36.6
25	25–29	0.936	0.906	965.4	34.6	26.2–43.0
30	30–34	0.914	0.887	970.8	29.2	22.7–35.8
35	35–39	0.883	0.851	964.2	35.8	27.9–43.7
40	40–44	0.843	0.805	953.2	46.8	36.4–57.3
45	45–49	0.787	0.742	939.5	60.5	44.9–76.2
			$_{35}q_{15}$ (0–4y) = 228.8‰ (95% CI: 205.2–252.5)			

### Indirect estimates derived from changes in the proportion of adult siblings surviving between two surveys

Recent changes in the proportions of adult siblings remaining alive can also be constructed for a cohort of respondents in two successive inquiries separated by about five years. This approach circumvents the need for an additional question to identify recent deaths but requires that changes in the proportion of adult siblings surviving between two surveys conducted at an awkward interval are converted into survivorship between conventional five-year age groups.

Except in late old age, adult human mortality rises as an exponential function of age and can be represented by a Gompertz-Makeham model. Moreover, most of the variation in mortality between populations is accounted for by $\alpha$, the level parameter of the model. The $\beta$, or shape, parameter of the model varies little between populations, even when they have very different levels of mortality [40]. This empirical finding provides the basis for a method for converting measures of cohort survivorship in between two surveys of a population conducted at an awkward interval into conventional measures of five-year survivorship. In essence, one can use an estimate of $\beta$ to interpolate within, or extrapolate from, estimated survival over age intervals equivalent to the inter-survey interval to survival over a five-year age interval (see Onine Appendix A.4).

Table 6 provides an example calculation based on the data from the 2015 Zimbabwe DHS, and the 2010–11 DHS, which was conducted an average of 4.76 years earlier. Proportions of adult siblings still alive at the time of the surveys are combined using the ratio $\frac{_{5}S^{15+}_{n-0.24}(t)}{_{5}S^{15+}_{n-5}(t-4.76)}$, where *t* represents the time of the 2015 survey. After a slight adjustment to account for the interval between surveys being just under 5 years, the ratio is converted into survival probabilities using the coefficients listed in Table 4. These probabilities are then chained together to produce the probability $_{35}q_{15}$, which is 236.1‰. This mortality estimate is only 4% lower than the probability obtained directly, which serves as our reference (245.4‰). While this method of combining data from two surveys seems promising, calculating confidence intervals around the ratios becomes more complex. Given the broader set of results detailed below, we will only present the point estimates here.

**Table 6:** Indirect calculation of age-specific mortality rates using proportions of adult siblings who are still alive at the time of the survey in the 2011 and 2015 Zimbabwe DHS, female mortality

n	Age group	DHS	DHS	Ratio	Adjusted ratio	$_{5}p_{n}$ (%)
		2010–11	2015			
		$_{5}S^{15+}_{n-5}$	$_{5}S^{15+}_{n-0.24}$			
		$$(t-4.76)$$	(*t*)			
					Adjustment = − 0.0551	
15	15–19	0.957	0.962	0.962	0.961	962.3
20	20–24	0.929	0.946	0.988	0.988	984.4
25	25–29	0.910	0.908	0.977	0.976	973.4
30	30–34	0.887	0.889	0.977	0.976	975.8
35	35–39	0.832	0.853	0.962	0.960	960.1
40	40–44	0.802	0.805	0.968	0.966	965.0
45	45–49	0.774	0.741	0.924	0.919	916.3
				$_{35}q_{15}$ (intersurvey period) = 236.1‰		

Note: the intersurvey period is 4.76 years. See Online Appendix A.4 for the calculation of the adjustment to the ratio

## Results

### Indirect estimates derived from adult lifetime proportions or changes in proportions surviving calculated from a single survey

In this section, we analyze all DHS data with an SSH module available at the time of writing. We first assess the performance of the two indirect methods requiring a single survey, based either on adult lifetime proportions or on recent changes in the proportions surviving. We compare our indirect estimates with the direct estimates. While the direct estimates serve as our benchmark, it is important to acknowledge that these might be affected by omission or misplacement of deaths, as well as inaccuracies in age reporting. A record linkage study conducted in the Niakhar Health and Demographic Surveillance System (in Senegal) revealed that respondents tended to underestimate the ages of living siblings, ages at the time of death, and the time elapsed since the deaths [41]. These reporting inaccuracies introduced downward biases in mortality estimates, although recent estimates (0–6 years before the survey) remained unaffected. Several studies also showed that under-reporting of deaths was more pronounced for deaths occurring further in the past [4, 13, 42]. Consequently, direct estimates cannot be regarded as a gold standard, even though those for the period immediately preceding data collection are more reliable. For these reasons, we also compare sibling estimates with those of the WPP (2024 Revision), which partly rely on DHS data but incorporate additional survey and census data [11]. The WPP also factor in the expected relationship between child and adult mortality, although this is largely informed by the historical record in high-income countries.

**Fig. 1:**
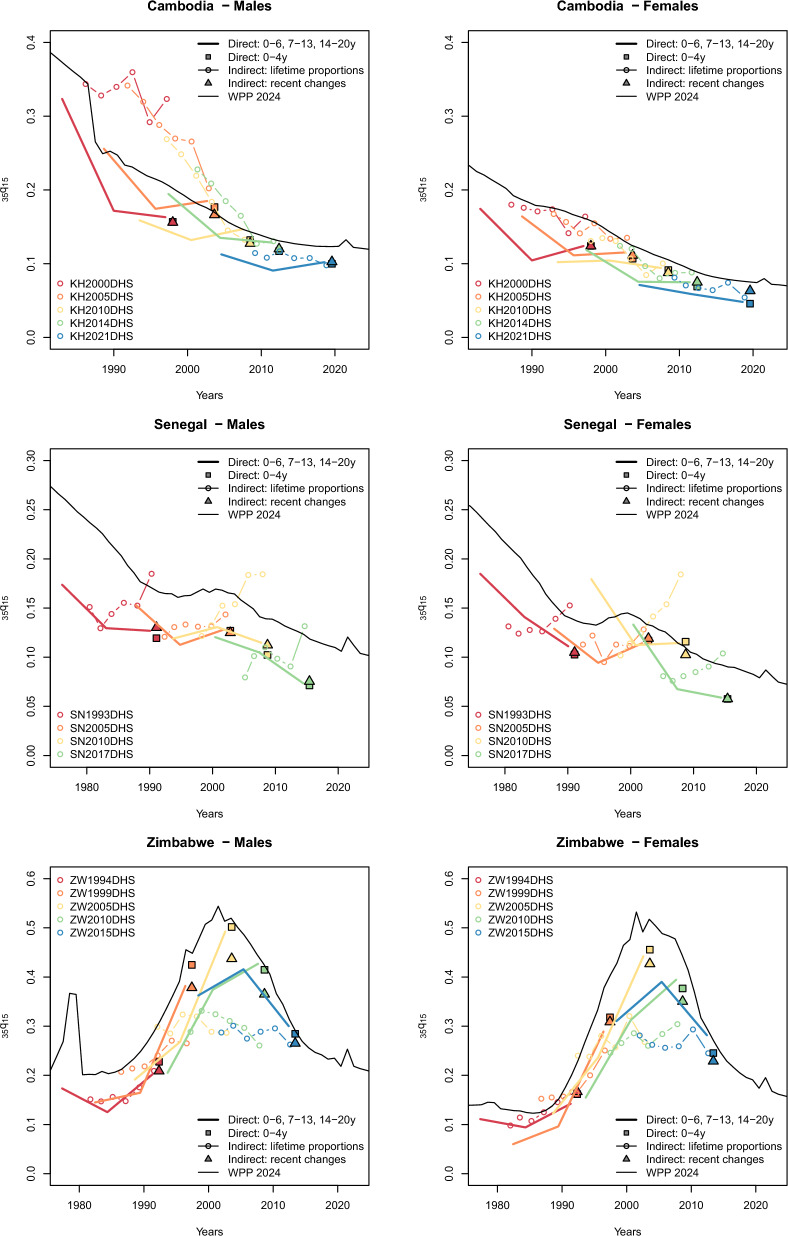
Estimates of the probability of dying between ages 15 and 50 ($_{35}q_{15}$) from SSH data using different methods in Cambodia, Senegal and Zimbabwe. Note: one set of direct estimates is calculated for periods of 0–6, 7–13, and 14–20 completed years before the survey (solid lines). Another set is calculated for the period 0–4 completed years before the survey (squares) to compare with indirect estimates derived from recent changes in proportions (triangles). The y-axis scale differs between panels of the figure

Figure 1 displays a series of $_{35}q_{15}$ probability estimates for Cambodia, Senegal and Zimbabwe. These three countries were chosen as examples as they have several surveys with sibling histories, and represent diverse mortality patterns. In Cambodia, the direct estimates are relatively consistent across surveys and indicate that the $_{35}q_{15}$ probability decreased by more than threefold from the 1980s to the end of the 2010s (e.g. from 323‰ in 1996 to 102‰ in 2018 for males). Trends inferred from adult lifetime proportions suggest a more gradual decline in mortality, although the mortality levels for the 1990s appear implausibly high among men. Indirect estimates derived from changes in the proportion surviving are virtually identical to those computed from full SSH for the 5 years preceding each survey. In Senegal, the direct estimates remain relatively consistent across surveys, although they are below the WPP values. Indirect estimates obtained from adult lifetime proportions are quite erratic, with several surveys indicating an increase in mortality. Indirect estimates from recent changes in the proportions surviving align closely with those derived from full SSH. Finally, in Zimbabwe, trends have been severely disrupted by the HIV/AIDS epidemic. Direct estimates suggest a fourfold increase in the $_{35}q_{15}$ probability between the early 1980s and the early 2000s. Indirect estimates from adult lifetime proportions appear too low: among men, they peak at 265.7‰, compared to a peak of 492.5‰ in direct estimates (in 2002). To a lesser extent, recent levels obtained indirectly from changes in the proportions surviving are also lower than direct estimates at the height of the epidemic.

**Fig. 2:**
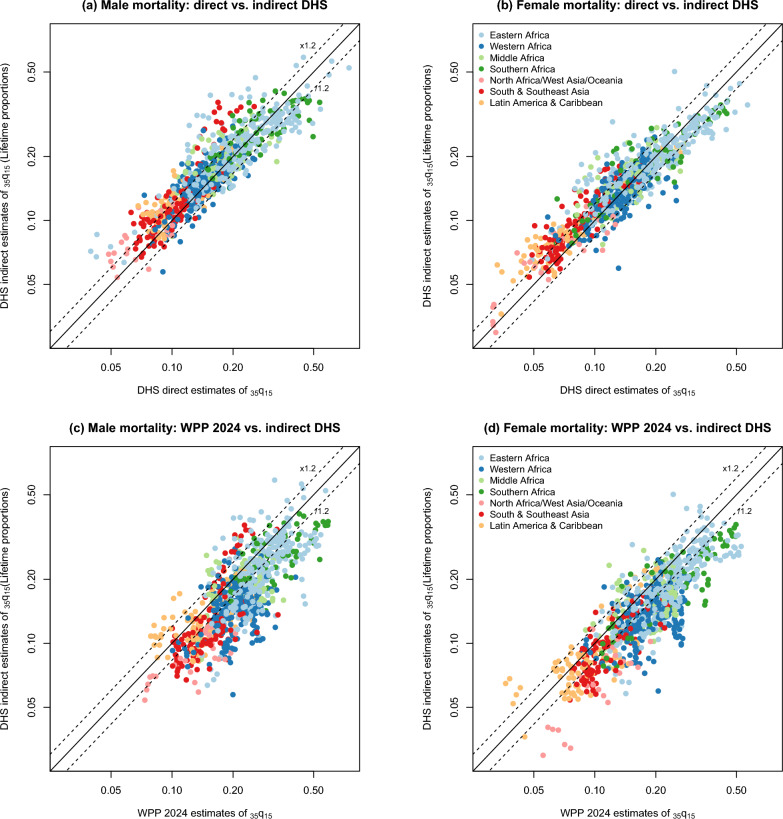
Indirect estimates of the probability of dying between ages 15 and 50 ($_{35}q_{15}$) obtained from adult lifetime proportions (0–15 years before data collection), compared to direct estimates from full SSH and the 2024 World Population Prospects

Figure 2 compares, for all DHS, the indirect estimates derived from adult lifetime proportions with the direct estimates from full SSH (upper panel) and the World Population Prospects 2024 (lower panel) [11]. We interpolated between direct estimates and between WPP values to obtain a reference for each indirect point estimate. These series are not fully consistent; if direct estimates are taken as a reference, the mean absolute percentage error (MAPE) is 18% for male mortality and 17% for female mortality. The indirect method based on adult lifetime proportions tends to provide higher mortality rates; the median ratio between indirect and direct estimates is 1.04 for males and 1.02 for females (Table 7). Although these ratios are close to one, these differences are significant for both sexes (p < 0.05, Wilcoxon signed-rank tests). The median ratios of indirect/direct estimates differ by region, being lowest in Eastern and Southern Africa (0.98 and 0.99), and highest in Latin America and the Caribbean (1.16) and South and Southeast Asia (1.16), two regions where adult mortality is relatively low and sibship sizes are smaller. Deviations are greater when indirect estimates from adult lifetime proportions are compared with mortality rates from the WPP. The MAPE values are 25% for males and 24% for females. The $_{35}q_{15}$ probabilities calculated from summary sibling histories tend to be much lower than in the WPP, with median ratios at 0.78 in males and 0.79 in females (Table 7). The median ratios decline with the age of respondents to reach 0.70 when estimates are derived from reports of respondents aged 45–49. By region, median ratios are lowest in West Africa, and in the few surveys conducted with sibling histories in North Africa, West Asia and Oceania. The pattern observed in West Africa is consistent with earlier comparisons made with previous revisions of the WPP [5, 42, 43] and these low ratios could be attributed either to poorer data quality in these regions or to systematic over-estimation of adult mortality in the WPP.

**Table 7:** Median ratios of indirect estimates of adult mortality from (a) adult lifetime proportions or (b) changes in proportions surviving, over direct estimates from SSH (upper panel) or WPP estimates (lower panel)

	(a) Adult lifetime proportions		(b) Change in proportions surviving	
	Median ratio	IQR	Median ratio	IQR
Compared with direct estimates from SSH	Ratios of $_{35}q_{15}$		Ratios of $_{35}q_{15}$	
Sex of siblings				
Males	1.04	0.93–1.20	0.97	0.93–﻿1.02
Females	1.02	0.91–1.18	0.98	0.94–﻿1.02
Region				
South & Southeast Asia	1.16	1.06–1.30	1.04	0.99–﻿1.09
Middle Africa	1.01	0.90–1.13	0.97	0.95–﻿1.00
Eastern Africa	0.98	0.88–1.14	0.94	0.92–﻿0.97
Southern Africa	0.99	0.84–1.19	0.94	0.91–﻿1.00
Western Africa	1.01	0.92–1.12	0.98	0.95–﻿1.02
Latin America & Car.	1.16	1.02–1.32	1.02	0.97–﻿1.07
North Africa/West Asia/Oceania	1.13	0.95–1.28	1.06	1.02–﻿1.09
Age group of respondents	Ratios of $_{35}q_{15}$		Ratios of $_{5}q_{n}$	
15–19	–	–	1.09	0.90–﻿1.42
20–24	1.05	0.91–1.25	1.26	1.06–﻿1.54
25–29	1.04	0.93–1.18	1.08	0.94–﻿1.26
30–34	1.02	0.94–1.17	0.85	0.74–﻿1.01
35–39	1.08	0.94–1.23	0.91	0.79–﻿1.09
40–44	1.03	0.92–1.18	0.90	0.77–﻿1.02
45–49	0.99	0.88–1.13	1.00	0.87–﻿1.18

Compared with WPP 2024	Ratios of $_{35}q_{15}$		Ratios of $_{35}q_{15}$	
Sex of siblings				
Males	0.78	0.67–﻿0.92	0.85	0.75–﻿0.98
Females	0.79	0.68–﻿0.94	0.84	0.73–﻿0.96
Region				
South & Southeast Asia	0.82	0.71–﻿0.96	0.85	0.80–﻿0.92
Middle Africa	0.86	0.73–﻿1.01	1.00	0.86–﻿1.06
Eastern Africa	0.80	0.69–﻿0.93	0.88	0.78–﻿0.99
Southern Africa	0.74	0.65–﻿0.90	0.86	0.81–﻿0.92
Western Africa	0.72	0.63–﻿0.83	0.77	0.69–﻿0.86
Latin America & Car.	0.88	0.75–﻿1.09	0.86	0.77–﻿1.01
North Africa/West Asia/Oceania	0.64	0.53–﻿0.77	0.64	0.53–﻿0.74
Age group of respondents	Ratios of $_{35}q_{15}$		Ratios of $_{5}q_{n}$	
15–﻿19	–	–	0.97	0.76–﻿1.31
20–﻿24	0.92	0.77–﻿1.10	0.96	0.74–﻿1.25
25–﻿29	0.85	0.73–﻿0.98	0.88	0.72–﻿1.06
30–﻿34	0.79	0.68–﻿0.94	0.77	0.64–﻿0.95
35–﻿39	0.76	0.67–﻿0.88	0.80	0.65–﻿0.92
40–﻿44	0.74	0.63–﻿0.85	0.78	0.65–﻿0.95
45–﻿49	0.70	0.60–﻿0.81	0.79	0.65–﻿0.94

Note: for indirect estimates computed from adult lifetime proportions, all ratios refer to the probability $_{35}q_{15}$, while for estimates derived from changes in proportions surviving, the median ratios computed by age groups refer to age-specific mortality from age 15 to 49

**Fig. 3:**
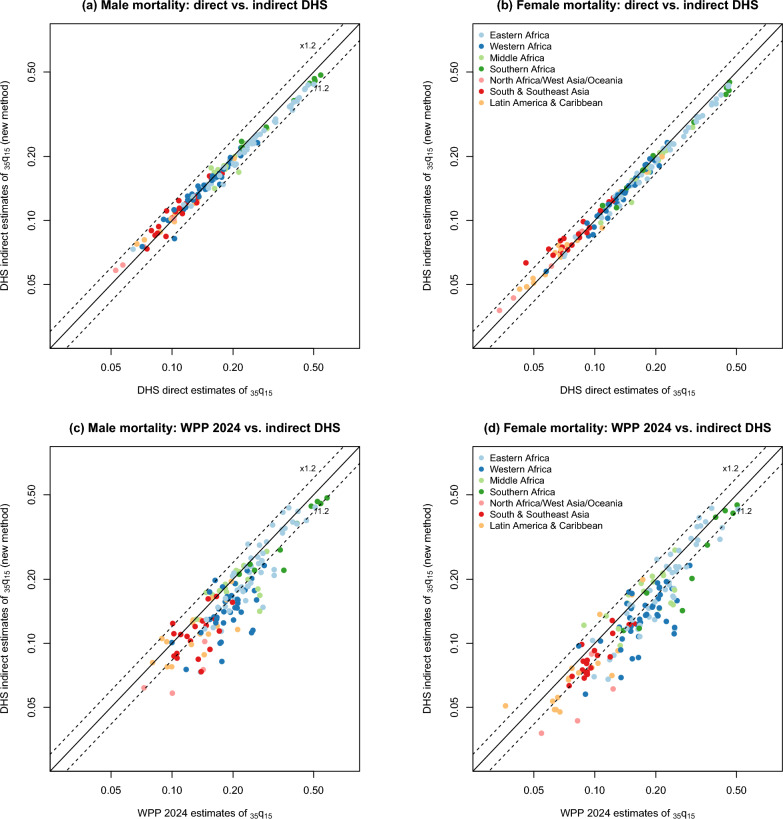
Indirect estimates of the probability of dying between ages 15 and 50 $_{35}q_{15}$ probability obtained from changes in proportions surviving (0–4 years before data collection), compared to direct estimates from full SSH and the 2024 World Population Prospects

Figure 3 compares the indirect estimates obtained using the new method based on changes in proportions surviving extracted from a single survey to those derived from direct calculations or data from the WPP. This comparison is also detailed in the bottom panel of Table 7. Each of the 154 surveys yields only one point estimate in this series. We observe a high level of consistency between the indirect and direct estimates, with a mean percentage error of 6% for both men and women. The median ratio for the summary probability 35*q*15 is 0.97 (IQR: 0.93$-$1.02) for brothers and 0.98 (IQR: 0.94$-$1.02) for sisters, and according to Wilcoxon signed-rank tests, these are not significantly different from 1. In all surveys except one for men (Zimbabwe 2005, shown in Fig. 1), the 95% confidence intervals around the $_{35}q_{15}$ probability obtained directly overlap with those calculated around the indirect estimate. Regional disparities remain modest and there is good agreement observed in West Africa. This suggests that age or dating errors in full SSH are not more pronounced in this region compared to others.

However, when the new indirect estimates are compared to recent mortality risks estimated in the WPP, consistently lower values are observed again in the DHS. The median ratios of DHS/WPP estimates are slightly higher than with adult lifetime proportions, at 0.85 (IQR: 0.75$-$0.98) for men and 0.84 (IQR: 0.73$-$0.96) for women (Table 7). These ratios are particularly low in West Africa and in surveys conducted in North Africa, Western Asia, and Oceania.

**Fig. 4:**
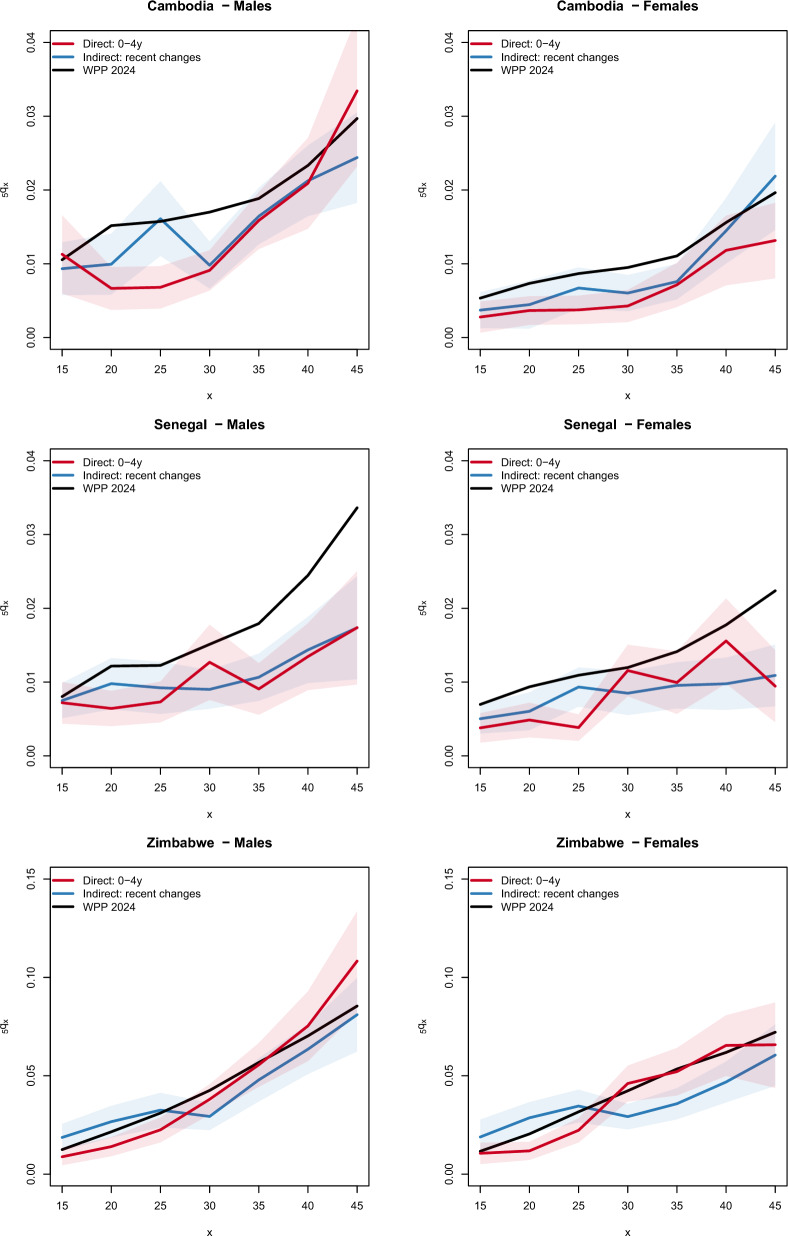
Age-specific mortality ($_{5}q_{x}$) in adults aged 15–49 based on the latest DHS with sibling histories in Cambodia (2021–2022), Senegal (2017) and Zimbabwe (2015), using the direct calculation and the method based on recent changes in proportions surviving, compared to WPP 2024 estimates. The y-axis scale differs between panels of the figure

With the method of Timæus et al. [18], the mortality levels inferred from different age groups of respondents correspond to varying reference periods, providing an overview of trends (assuming they have been linear), but not allowing for the reconstruction of age-specific mortality patterns. In contrast, with the new method focused on recent deaths, only mortality from the last five years is estimated. However, in addition to the synthetic probability $_{35}q_{15}$, this method also allows for the examination of age-specific patterns. This is illustrated for Cambodia, Senegal, and Zimbabwe in Fig. 4, where the estimates are compared with those from the United Nations and estimates calculated directly from the latest DHS for each country. Except for a few cases where the differences between the two sets of estimates are significant, the age-specific probabilities estimated using the direct and indirect methods are consistent. When we compare the direct and indirect estimates from the DHS with the WPP, there is good consistency in Zimbabwe, but the WPP predicts a faster rise in mortality with age in Senegal.

A more systematic examination of age-specific mortality is provided in Table 7, with ratios of indirect to direct estimates calculated for risks of dying per five-year interval on the 154 DHS. Median ratios are higher than 1 up to age 30, followed by lower ratios between ages 30 and 45. It is difficult to establish whether these fluctuations reflect errors in age reporting affecting direct estimates, or biases inherent in the model used for indirect estimation. Estimates based on changes in the proportion of siblings surviving are also compared with UN estimates in the bottom panel of Table 7. Again, ratios decline with age, from 0.97 for $_{5}q_{15}$ to 0.79 for $_{5}q_{45}$. This could be attributed to a number of factors, including bias in the coefficients used for indirect estimation, more pronounced reporting issues among older respondents in the DHS, or deviations between the actual age-specific patterns and those reconstructed in the WPP.

### Indirect estimates derived from changes in the proportion of adult siblings surviving between two surveys

**Fig. 5:**
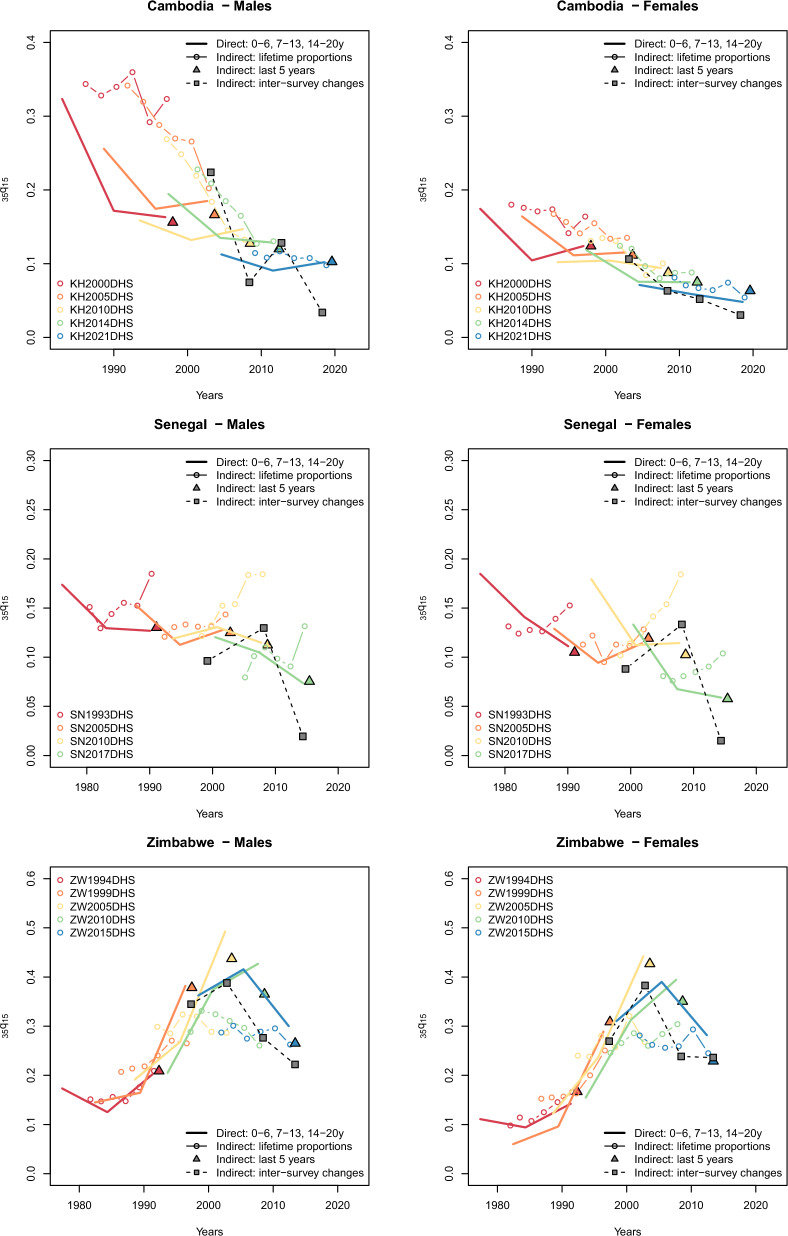
Estimates of the probability of dying between ages 15 and 50 ($_{35}q_{15}$) based on the full SSH, on lifetime survival of adult siblings, on the survival to survey of cohorts of siblings alive 5 years earlier, and on cohort changes in sibling survival in between successive surveys. The y-axis scale differs between panels of the figure

We now present results based on the combination of two inquiries, to assess the potential of this approach in cases where the question identifying recent deaths was not asked. Figure 5 presents estimates of adult mortality for three countries based on inter-survey changes in the proportions that remain alive of the adult siblings of cohorts of respondents. The intervals between the surveys range from a bit less than 4 years to a bit more than 7 years, with the exception of those for 1993–2005 in Senegal, which should probably be discounted. The inter-survey estimates are compared with results from other sibling-based estimation methods that were presented initially in Figure 1. In Cambodia, the estimates of women’s mortality are close to those obtained by asking about deaths of adult siblings during the 5 years before the survey. The estimates of men’s mortality, however, follow a sawtooth pattern, with only those for the interval between the 2010 and 2014 coinciding with the results from the other methods. This pattern of results might result from fluctuations in the completeness of reporting of dead brothers from survey to survey, with the data for 2000 and 2021 being least complete and those for 2005 and 2014 perhaps most complete. In Senegal, the estimates for both men and women are erratic. Those for 2005–11 seem somewhat high and those for 2011–17 seem too low, suggesting that the reporting of dead siblings may have been most complete in 2011. In Zimbabwe, all the estimates are lower than those from those obtained from data on the survival of siblings during the 5 years before the survey except for that based on cohort changes in the proportions of respondents adult sisters that are alive in between the 2011 and 2015 surveys. This pattern of results might result from a steady improvement over time in the quality of reporting on dead siblings.

## Discussion

In countries without reliable vital statistics, the inclusion of sibling histories in several survey programs made a crucial contribution to our understanding of adult mortality trends. More than 150 Demographic and Health Surveys have incorporated detailed questions about siblings, prompting respondents about the ages of surviving siblings, or, ages at death and the timing of deaths of those who died. Full SSH are an irreplaceable source of information, allowing comprehensive tests of data quality [44], and the detailed modelling of mortality trends, sex ratios and age patterns of adult mortality [4, 43]. Because of the time required for data collection, however, the full SSH module is not systematically included in all DHS and MICS, and it is not well suited to censuses or rapid turnaround surveys such as those conducted in complex humanitarian emergencies [45].

In this study, we evaluated the performance of a new indirect estimation method requiring only summary SSH. Compared with the series of questions initially proposed by Timæus and colleagues in 2001 [18], only one additional question is needed to identify recent deaths. The method can therefore be combined with the one based on adult lifetime proportions of surviving siblings, in contexts where mortality trends have been regular. Using these data, analysts can obtain both a trend (with the original coefficients) and a recent estimate (with the method proposed here).

When applied to microsimulations, the original indirect method based on adult lifetime proportions provides unbiased estimates of adult mortality (see Online appendix A.3). However, indirect estimates calculated from adult lifetime proportions in DHS data are systematically higher than direct mortality rates. There are several possible explanations for this. First, it could be related to the conversion of age-specific rates into the summary index $_{35}q_{15}$ using model life tables. In countries affected by HIV, using an age pattern affected by AIDS would reduce slightly the estimates of $_{35}q_{15}$. Second, direct estimates may be too low due to underestimation of the age of living siblings, ages at death, and the time since deaths [41, 46]. Third, the age patterns of fertility and mortality in survey data could differ from those used to compute the coefficients allowing to convert proportions into survivorship probabilities. In particular, in both the analytical computations and microsimulations, all women are exposed to the same fertility distribution, regardless of their parity and the interval since the last birth (apart from a minimal birth interval). It might be possible to improve the conversion of proportions of surviving siblings to risks of dying by adding some predictors, such as an indicator of the dispersion of the fertility distribution, and this is an area for future research.

Two important limitations of the original indirect method will inevitably remain. First, estimates derived from adult lifetime proportions refer to a relatively distant past (3–15 years before the survey), and second, trends in mortality are assumed to be regular. To address these limitations, we introduced a new indirect method focusing on recent deaths. When applied to survey data, this method provided estimates that were highly consistent with those derived from the full SSH module. The mean percentage error between direct and indirect estimates was only 6% for both sexes, and the median ratios for the summary probability $_{35}q_{15}$ were close to one (0.97 (IQR: 0.93$-$1.02) for brothers and 0.98 (IQR: 0.94$-$1.02) for sisters). We detected significant differences between the two sets of $_{35}q_{15}$ values in only one survey out of 154 (and for male mortality only). When the length of the interview needs to be reduced, and the focus is on recent mortality, this indirect method provides a good alternative to the full SSH module. In addition to saving time, indirect estimates will be less sensitive to errors in the ages of siblings, their ages at death, and the timing of their deaths than direct estimates. The role of models in the estimation process is reduced because there is no need to extrapolate age-specific probabilities to the $_{35}q_{15}$ index using standard age patterns; they can be directly chained together. The method is applicable to countries affected by conflicts and epidemics, as it does not require the assumption that trends have been regular and unidirectional. Finally, the method makes it possible to measure age-specific mortality, although the reason why these estimates yield a different age pattern of mortality from the direct ones needs to be investigated further.

The method is, however, reliant on the correct attribution of deaths to the last five years before the interview date and the accurate reporting of the age of respondents. Consistency tests should be carried out before applying it to new survey data: analysts could, for example, examine the plausibility of the number of siblings who have reached the age of 15, in light of DHS surveys carried out previously in similar settings, the plausibility of the number of deceased siblings, and compare the proportions of recent deaths with those reported in full sibling histories. It is also necessary to check what proportion of respondents did not answer the questions or did not know how many siblings had reached the age of 15, or how many had died. In cases where both males and females have been asked about their siblings, it is useful to compare the answers given by male and female respondents [47]. For example, misreporting of respondents’ ages may be suspected if the reported proportions of surviving siblings differ significantly between male and female respondents.

We also showed that recent changes in the proportions of adult siblings remaining alive can be constructed for a cohort of respondents in two successive inquiries separated by about five years. It is possible to adjust data from surveys conducted at awkward intervals to refer to conventional 5-year age groups. However, the resulting estimates were more erratic than the other estimates made from data on sibings, presumably because of differences in sample characteristics, changes in the size and composition of sibships, selective mortality of respondents and/or different rates of misreporting. Nevertheless, in applications in which no questions on the timing of deaths have been asked, this approach may be the only way to obtain an up-to-date estimate of mortality from the summary data on siblings. In other applications, moreover, it may represent a useful cross-check on the other estimates and provide additional insights into the quality of the data.

Given the importance of sibling histories in the measurement of adult mortality worldwide, further research is needed on data quality, possible biases and estimation methods. Other techniques for quantifying mortality from summary data are also conceivable. For example, age differences between the respondent and her siblings could be imputed from a full sibling history collected in the past, or from regional distributions, as in the methods developed for under-five mortality [48, 49].

These future developments and the possibility of using the method proposed in this study should motivate the inclusion of summary questions on siblings in all DHS surveys and in other programs such as MICS. The set of questions on the number of siblings who reached age 15, the number who have died since and the number of recent deaths could also be integrated into surveys where the duration of interviews needs to remain short, such as in mortality surveys conducted in complex emergencies or through mobile phones [50]. The global burden of mortality in early adulthood is currently concentrated in countries where the development of civil registration systems remains slow. It is therefore vital to improve the collection of data on these adults from surveys and censuses in the interim, pending full registration coverage.

## Supplementary Information

Supplementary file.

## Data Availability

The R code to re-create the set of 192 microsimulations and to reconstruct summary SSH within these simulations is available in a GitHub repository (https://github.com/brunomasquelier/SummarySSH). This repository also includes the code used to apply the method to Demographic and Health Surveys. CSV data files with age-specific mortality rates and estimates of _35_q_15_ from the direct and indirect approaches, derived from the 154 Demographic and Health Surveys are also available. The survey data are publicly available through https://www.dhsprogram.com/.

## Abbreviations

CDC: Centers for Disease Control and Prevention
DHS: Demographic and Health Surveys
GBD: Global Burden of Disease
MICS: Multiple Indicator Cluster Surveys
SSH: Sibling survival histories
WPP: World Population Prospects

## Footnotes

1 https://www.lshtm.ac.uk/research/centres-projects-groups/rapid-mortality-mobile-phone-survey.

2 https://github.com/mrc-ide/demogsurv.

3 https://cran.r-project.org/web/packages/DHS.rates/index.html.

4 https://github.com/dfeehan/siblingsurvival.
