# Supplementary material for: A new method for estimating recent adult mortality from summary sibling histories
Source: Popul Health Metr. 2024 Nov 12;22:32. doi: 10.1186/s12963-024-00350-0 (PMC11555974; doi:10.1186/s12963-024-00350-0)
Supplement: Supplementary file 1 — Supplementary file. [file 12963_2024_350_MOESM1_ESM.pdf]

ADDITIONAL FILE

A new method for estimating recent adult mortality from summary sibling histories

Bruno Masquelier, Ashira Menashe-Oren, Georges Reniers, Ian M. Timæus *Population Health Metrics*, 10.1186/s12963-024-00350-0

A.1. Coefficients for the indirect calculation based on the number of adult siblings surviving after age 15

n	β_0	β_1	R^2	CV
25	-0.0003	1.0011	0.9818	0.0042
30	-0.1546	1.1560	0.9950	0.0034
35	-0.1645	1.1660	0.9981	0.0029
40	-0.1388	1.1406	0.9984	0.0035
45	-0.1140	1.1168	0.9985	0.0042
50	-0.1018	1.1066	0.9986	0.0052

Table A1 – *Coefficients used to convert proportions of surviving siblings (among those who survived to 15 years) to survival probabilities - Sc: [1]*

A.2. DHS surveys used in this study

Country	Year	Sample size (women aged 15-49)	Region
Afghanistan	2015	29461	South Asia
Angola	2015-16	14379	Middle Africa
Burkina Faso	1998-99	6445	Western Africa
Burkina Faso	2003	12477	Western Africa
Burkina Faso	2010	17087	Western Africa
Burkina Faso	2021	17659	Western Africa
Benin	1996	5491	Western Africa
Benin	2006	17794	Western Africa
Benin	2017-18	15928	Western Africa
Bolivia	1994	8603	South America
Bolivia	2003	17654	South America
Bolivia	2008	16939	South America
Brazil	1996	12612	South America
Burundi	2010	9389	Eastern Africa
Burundi	2016-17	17269	Eastern Africa
Congo Democratic Republic	2007	9995	Middle Africa
Congo Democratic Republic	2013-14	18827	Middle Africa
Central African Republic	1994-95	5884	Middle Africa
Congo	2005	7051	Middle Africa
Congo	2011-12	10819	Middle Africa
Cote d'Ivoire	1994	8099	Western Africa
Cote d'Ivoire	2005	5183	Western Africa
Cote d'Ivoire	2011-12	10060	Western Africa
Cote d'Ivoire	2021	14877	Western Africa
Cameroon	1998	5501	Middle Africa
Cameroon	2004	10656	Middle Africa
Cameroon	2011	15426	Middle Africa
Cameroon	2018	13527	Middle Africa
Colombia	2015	38718	South America
Dominican Republic	2002	23384	Caribbean
Dominican Republic	2007	27195	Caribbean
Ethiopia	2000	15367	Eastern Africa
Ethiopia	2005	14070	Eastern Africa
Ethiopia	2011	16515	Eastern Africa
Ethiopia	2016	15683	Eastern Africa
Gabon	2000	6183	Middle Africa
Gabon	2012	8422	Middle Africa
Gabon	2019-21	11043	Middle Africa
Gambia	2013	10233	Western Africa
Gambia	2019-20	11865	Western Africa
Guinea	1999	6753	Western Africa
Guinea	2005	7954	Western Africa
Guinea	2012	9142	Western Africa
Guatemala	1995	12403	Central America

Guatemala	2014-15	25914	Central America
Haiti	2000	10159	Caribbean
Haiti	2005-06	10757	Caribbean
Haiti	2016-17	14371	Caribbean
Indonesia	1994	28168	Southeast Asia
Indonesia	1997	28810	Southeast Asia
Indonesia	2002-03	29483	Southeast Asia
Indonesia	2007	32895	Southeast Asia
Indonesia	2012	45607	Southeast Asia
Jordan	1997	5548	West Asia
Kenya	1998	7881	Eastern Africa
Kenya	2003	8195	Eastern Africa
Kenya	2008-09	8444	Eastern Africa
Kenya	2014	31079	Eastern Africa
Cambodia	2000	15351	Southeast Asia
Cambodia	2005	16823	Southeast Asia
Cambodia	2010	18754	Southeast Asia
Cambodia	2014	17578	Southeast Asia
Cambodia	2021-22	19496	Southeast Asia
Comoros	2012	5329	Eastern Africa
Liberia	2007	7092	Western Africa
Liberia	2013	9239	Western Africa
Liberia	2019-20	8065	Western Africa
Lesotho	2004	7095	Southern Africa
Lesotho	2009	7624	Southern Africa
Lesotho	2014	6621	Southern Africa
Morocco	1992	9256	North Africa
Morocco	2003-04	16798	North Africa
Madagascar	1997	7060	Eastern Africa
Madagascar	2003-04	7949	Eastern Africa
Madagascar	2008-09	17375	Eastern Africa
Mali	1995-96	9704	Western Africa
Mali	2001	12849	Western Africa
Mali	2006	14583	Western Africa
Mali	2012-13	10424	Western Africa
Mali	2018	10519	Western Africa
Myanmar	2015-16	12885	Southeast Asia
Mauritania	2019-21	15714	Western Africa
Malawi	1992	4849	Eastern Africa
Malawi	2000	13220	Eastern Africa
Malawi	2004	11698	Eastern Africa
Malawi	2010	23020	Eastern Africa
Malawi	2015-16	24562	Eastern Africa
Mozambique	1997	8779	Eastern Africa
Mozambique	2003	12418	Eastern Africa
Mozambique	2011	13745	Eastern Africa
Nigeria	2008	33385	Western Africa

Nigeria	2013	38948	Western Africa
Nigeria	2018	41821	Western Africa
Niger	1992	6503	Western Africa
Niger	2006	9223	Western Africa
Niger	2012	11160	Western Africa
Namibia	1992	5421	Southern Africa
Namibia	2000	6755	Southern Africa
Namibia	2006-07	9804	Southern Africa
Namibia	2013	9176	Southern Africa
Nepal	1996	8429	South Asia
Nepal	2006	10793	South Asia
Nepal	2016	12862	South Asia
Peru	1991-92	15882	South America
Peru	1996	28951	South America
Peru	2000	27843	South America
Peru	2004-06	19090	South America
Peru	2007-08	22558	South America
Papua New Guinea	2016-18	15198	Pacific Islands
Philippines	1993	15029	Southeast Asia
Philippines	1998	13983	Southeast Asia
Rwanda	2000	10421	Eastern Africa
Rwanda	2005	11321	Eastern Africa
Rwanda	2010	13671	Eastern Africa
Rwanda	2014-15	13497	Eastern Africa
Rwanda	2019-20	14634	Eastern Africa
Sierra Leone	2008	7374	Western Africa
Sierra Leone	2013	16658	Western Africa
Sierra Leone	2019	15574	Western Africa
Senegal	1992-93	6310	Western Africa
Senegal	2005	14602	Western Africa
Senegal	2010-11	15688	Western Africa
Senegal	2017	16787	Western Africa
Sao Tome and Principe	2008-09	2615	Middle Africa
Eswatini	2006-07	4987	Southern Africa
Chad	1996-97	7454	Middle Africa
Chad	2004	6085	Middle Africa
Chad	2014-15	17719	Middle Africa
Togo	1998	8569	Western Africa
Togo	2013-14	9480	Western Africa
Timor-Leste	2009-10	13137	Southeast Asia
Timor-Leste	2016	12607	Southeast Asia
Tanzania	1996	8120	Eastern Africa
Tanzania	2004-05	10329	Eastern Africa
Tanzania	2010	10139	Eastern Africa
Tanzania	2015-16	13266	Eastern Africa
Tanzania	2022	15254	Eastern Africa
Uganda	1995	7070	Eastern Africa

Uganda	2000-01	7246	Eastern Africa
Uganda	2006	8531	Eastern Africa
Uganda	2011	8674	Eastern Africa
Uganda	2016	18506	Eastern Africa
South Africa	1998	11735	Southern Africa
South Africa	2016	8514	Southern Africa
Zambia	1996	8021	Eastern Africa
Zambia	2001-02	7658	Eastern Africa
Zambia	2007	7146	Eastern Africa
Zambia	2013-14	16411	Eastern Africa
Zambia	2018	13683	Eastern Africa
Zimbabwe	1994	6128	Eastern Africa
Zimbabwe	1999	5907	Eastern Africa
Zimbabwe	2005-06	8907	Eastern Africa
Zimbabwe	2010-11	9171	Eastern Africa
Zimbabwe	2015	9955	Eastern Africa

Table A2 – *Surveys included in this study, sample sizes and region*

Note: We did not have access to datasets of surveys conducted in Eritrea (1995), Mauritania (2000) and Yemen (1997 and 2013). We excluded the SSH data from Peru (2009, 2010, 2012) because of missing data on imputed dates of birth and dates of death. We also excluded surveys from Bolivia (1989), Dominican Republic (1996), Egypt (1988), Ghana (1993), India (1999, 2006), Pakistan (2006), Sudan (1990) because the SSH data was not in a standard format.

A.3. Calibration of the microsimulation set

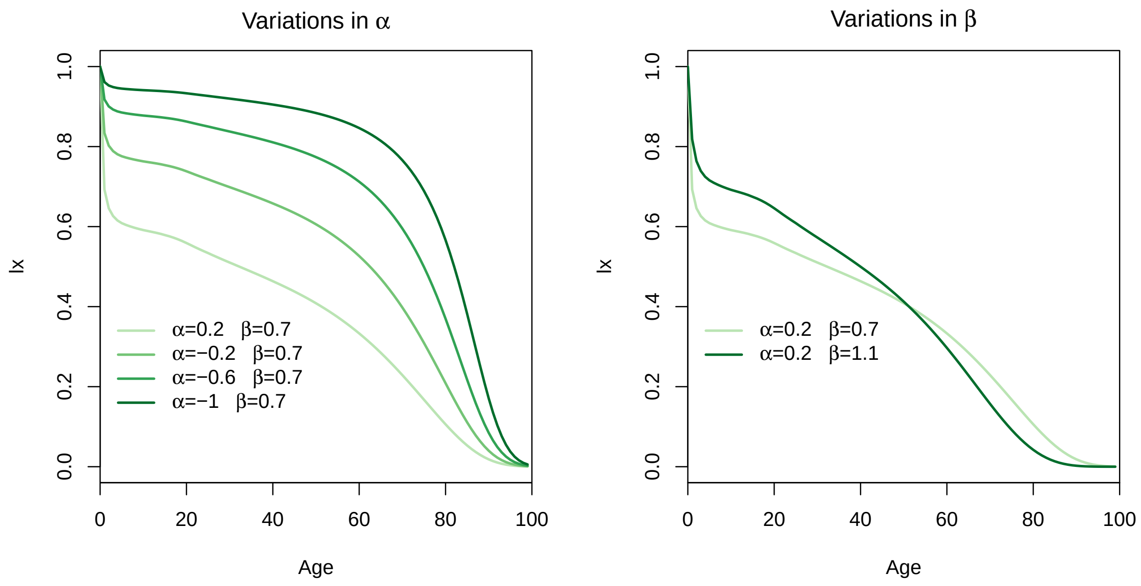

Figure A1 – *Age patterns of mortality introduced in the simulations, Brass General Standard [2]*

We calibrate the microsimulation model by calculating mortality in the simulated populations for the last 5 years and confronting these estimates with the life tables which were introduced as input parameters (Figure A3). As about 290 adult deaths (15-49) were produced over the last 5-year period in each simulation (IQR: 119-439), the recalculated ${}_{35}q_{15}$ probabilities are not identical to those in the input life tables, due to random fluctuations. However, these probabilities are unbiased. The median ratio between these probabilities and those in the input life tables is 1.00. The mean percentage deviation in ${}_{35}q_{15}$ is 5.4%. The coefficient of variation around ${}_{35}q_{15}$ is 6.8% on average. In 180 simulations (94%), the ${}_{35}q_{15}$ values from the input life tables are contained in the 95% confidence intervals.

By selecting only women aged 15-49 years at the end of the simulations and reconstructing their mother's birth history, mortality can be estimated directly from the full SSH, again for the last 5 years. Sibling-based estimates are included in Figure A3. About 466 recent adult deaths are mentioned in each set of SSH, which is higher than the actual number of deaths because most sisters are referenced multiple times. Despite this repetition of some sibships, the direct method based on full SSH provides unbiased estimates; the median ratio between the sibling-based and population-based probabilities ${}_{35}q_{15}$ is 1.00. The mean absolute percentage deviation of the sibling-based estimates is 5.8% when taking the population-based rates as reference. The coefficient of variation around the probability ${}_{35}q_{15}$ is 5.9% on average when computed from SSH. In 173 simulations (90%), the "true" probabilities calculated on the whole population are contained in the 95% confidence intervals around the sibling-based estimates.

We also applied the indirect method developed by Timæus et al. [1] to the microsimulated SSH. Figure A4 displays the probabilities ${}_{35}q_{15}$ obtained indirectly from reports of respondents aged 45-49, and the corresponding probabilities in the life table used to generate the simulations. The coefficients provide unbiased estimates of the risks of dying underlying these simulations. The median ratios of indirect to direct estimates of ${}_{n-15}q_{15}$ are all close to 1, and decline only slightly with age (1.02 in respondents aged 20-24 to 0.98 in respondents aged 45-49). Across all age groups, the mean absolute deviation between indirect estimates of ${}_{n-15}q_{15}$ and the corresponding input life table values is 8.5%.

A.4. Estimating survivorship between conventional five-year age groups from changes in the proportion of adult siblings surviving between two surveys conducted at an awkward interval

If two surveys have been conducted at an awkward interval, one can estimate the survivorship of the siblings of cohorts of respondents over that interval by tabulating the data from one of the surveys for unconventional age groups. This note suggests a method for working back from such cohort data to conventional measures of mortality. It requires that the results of the second survey are tabulated for unconventional age groups that correspond to conventional five-year cohorts in the first survey. For example, in the case of a 5.75 year interval between the inquiries, one can produce a series of estimates of ${}_5p_n$ from the following cohort measures of sibling survival:

$$\frac{{}_5S^{15+}_{n+0.75}(t)}{{}_5S^{15+}_{n-5}(t-5.75)} \tag{1}$$

The approach that we adopt is to adjust this ratio up or down so that it approximates to the proportion of the adult siblings of respondents aged $n-5$ to n that survive for exactly 5 years. Once this has been done, one can estimate mortality using the synthetic cohort methods proposed in this paper.

Define the gompit of life table survivorship by duration x from any base age n as:

$${}_xz_n = \ln(-\ln({}_xp_n)) \tag{2}$$

Gompertz survivorship is equally log mortality since $-\ln({}_xp_n) = x.{}_xm_n$.

The Gompertz-Makeham model describes mortality as the combination of an age-independent extrinsic component, λ, and an intrinsic component that is an exponential function of age:

$$\mu_z = \lambda + \alpha e^{\beta z} \tag{3}$$

Integrating this expression for μ_z between any two adult ages n and $n+x$:

$$-\ln({}_xp_n) = \lambda x + \frac{\alpha}{\beta}(e^{\beta x} - 1) \tag{4}$$

so that:

$${}_xz_n = \ln\left(\lambda x + \frac{\alpha}{\beta}(e^{\beta x} - 1)\right) \tag{5}$$

Systematic variation across populations in ${}_xz_n$ is captured largely by variations in extrinsic mortality, λ, and in the level or shape parameter of the Gompertz

model, α, which has a multiplicative effect on the mortality rates. The scale parameter β, which is equivalent to the slope of the log mortality schedule by age, varies far less between either populations or, Vaupel has suggested, individuals [4]. Instead, Vaupel hypothesizes, differences in population-level and individual frailty established prior to adulthood, that are captured by α, largely account for both differential mortality and the decelerating rate of increase in mortality with age in late old age due to the selection out of the population of its frailest members.

Expanding the right-hand side of this expression for Gompertz survivorship as a Puiseux series:

$$ {}_x z_n = \ln\left((\alpha+\lambda)x\right) + \frac{\alpha\beta}{2(\alpha+\lambda)}x + \frac{\alpha\beta^2(\alpha+4\lambda)}{24(\alpha+\lambda)^2}x^2 \ldots \tag{6} $$

where the terms in x^2 and higher powers of x can usually be ignored. Thus, Gompertz survivorship at duration x, offset by log duration, is an approximately linear function of duration:

$$ {}_x z_n - \ln(x) \approx a + bx \tag{7} $$

where $a = \ln(\alpha+\lambda)$ and $b = \alpha\beta/2(\alpha+\lambda)$. For baseline ages, n, at which intrinsic mortality, α, is considerably greater than extrinsic mortality, λ, the slope parameter b will approach 0.5β. Thus, in any population i:

$$ {}_x z_n(i) - \ln(x) \approx a(i) + bx \tag{8} $$

with b, like β, remaining close to constant across a wide range of baseline ages, n, life expectancies at birth, the two sexes, and different families of model life tables.

If this model fits data on human mortality adequately, it provides the basis for a straightforward way of estimating five-year survivorship from data on lifetime survivorship collected at awkward intervals. If the interval separating the surveys is x years and the survivorship of the siblings of cohorts of respondents has been estimated by tabulating the data from the second survey for unconventional age groups:

$$ {}_x \hat{z}_n(i) = {}_x z_n(i) - \left(\ln\left(\frac{x}{5}\right) + \beta(x-5)\right). \tag{9} $$

For example, if two inquiries have been conducted 5 years 9 months apart, one can estimate the gompits of the five-year survivorship probabilities by subtracting $0.14 + 0.75\beta$ from the gompits of survivorship over the interval of 5.75 years.

While this relationship applies in the life table, because:

$$ {}_5p_n \approx \frac{{}_5S_n^{15+}}{{}_5S_{n-5}^{15+}} \tag{10} $$

one will obtain almost the same final estimate by applying the adjustment to the ratio of the proportions of their adult siblings remaining alive reported by a cohort of respondents in two successive inquiries and then predicting ${}_5p_n$ as one would by adjusting an estimate of ${}_xp_n$ directly.

We estimated β for ages, n, of 20 to 45 years at five-year intervals by fitting a regression model to the four regional families of Princeton model life tables by sex with life expectancies at birth of 50, 60, 70, and 80 years. For each n, we calculated ${}_3p_{n-5}$, ${}_4p_{n-5} \ldots {}_7p_{n-5}$ to estimate how survival from a conventional five-year age group into an unconventional one over an awkward interval relates to a conventional ratio. We then predicted ${}_xz_n$ from age, life expectancy at birth (LEB), sex and their first-order interactions, treated as factor variables, and the duration of exposure, x, measured at integer durations of 3 to 7 years but treated as a continuous variable:

$$ {}_xz_n - \ln(x) = \mathrm{Age}_i + \mathrm{Sex}_j + \mathrm{LEB}_k + \mathrm{Age}_i.\mathrm{Sex}_j + \mathrm{Age}_i.\mathrm{LEB}_k + \mathrm{Sex}_j.\mathrm{LEB}_k + \beta x \tag{11} $$

As one would predict, based on our discussion of the Gompertz-Makeham model of mortality, the results suggest that x is close to linearly related to ${}_xz_n - \ln(x)$. Neither including a quadratic term in x nor estimating the effect of duration using a series of dummy variables for x significantly improves the fit of the model. Moreover, as Vaupel posited [4], inspection of the significance of the coefficients of the interaction terms in an exhaustive set of models, together with their log-likelihoods and AICs, fails to provide any evidence that β varies systematically by age, sex, life expectancy or regional family of Princeton model life tables. Table A3 presents the β coefficient and indicates the extent to which its estimated value varied across different subgroups of life tables.

Thus, in the case of two surveys conducted 5.75 years apart, the adjustment to be subtracted from the gompits of the proportions of siblings surviving between the surveys to obtain measures for survivorship between conventional five-year age groups is $0.14 + 0.025 \times (5.75 - 5) = 0.159$. For two surveys conducted 4.25 years apart with the data from the second survey tabulated for unconventional age groups starting at $n - 0.75$, one would subtract $-0.1625 + 0.025 \times (4.25 - 5) = -0.181$, which is to say add 0.181.

Sub-groups for which values of β are predicted	β for adjusting sibling survival into an unconventional age group $_xz_n = \ln\left(-\ln\left(\frac{_5S^{15+}_{n-5+x}(t)}{_5S^{15+}_{n-5}(t-x)}\right)\right) - \left(\ln\left(\frac{x}{5}\right) + \beta(x-5)\right)$
Overall	0.025
e_0 (50,60,70,80)	0.018 — 0.031
Initial age (n = 20 to 45, by 5)	0.012 — 0.046
Sex	0.023 — 0.026
Princeton regional family	0.020 — 0.026

Table A3 – *Adjustment factor (β) for converting survivorship of siblings over awkward durations, x, of between 3 and 7 years to survivorship for exactly 5 years.*

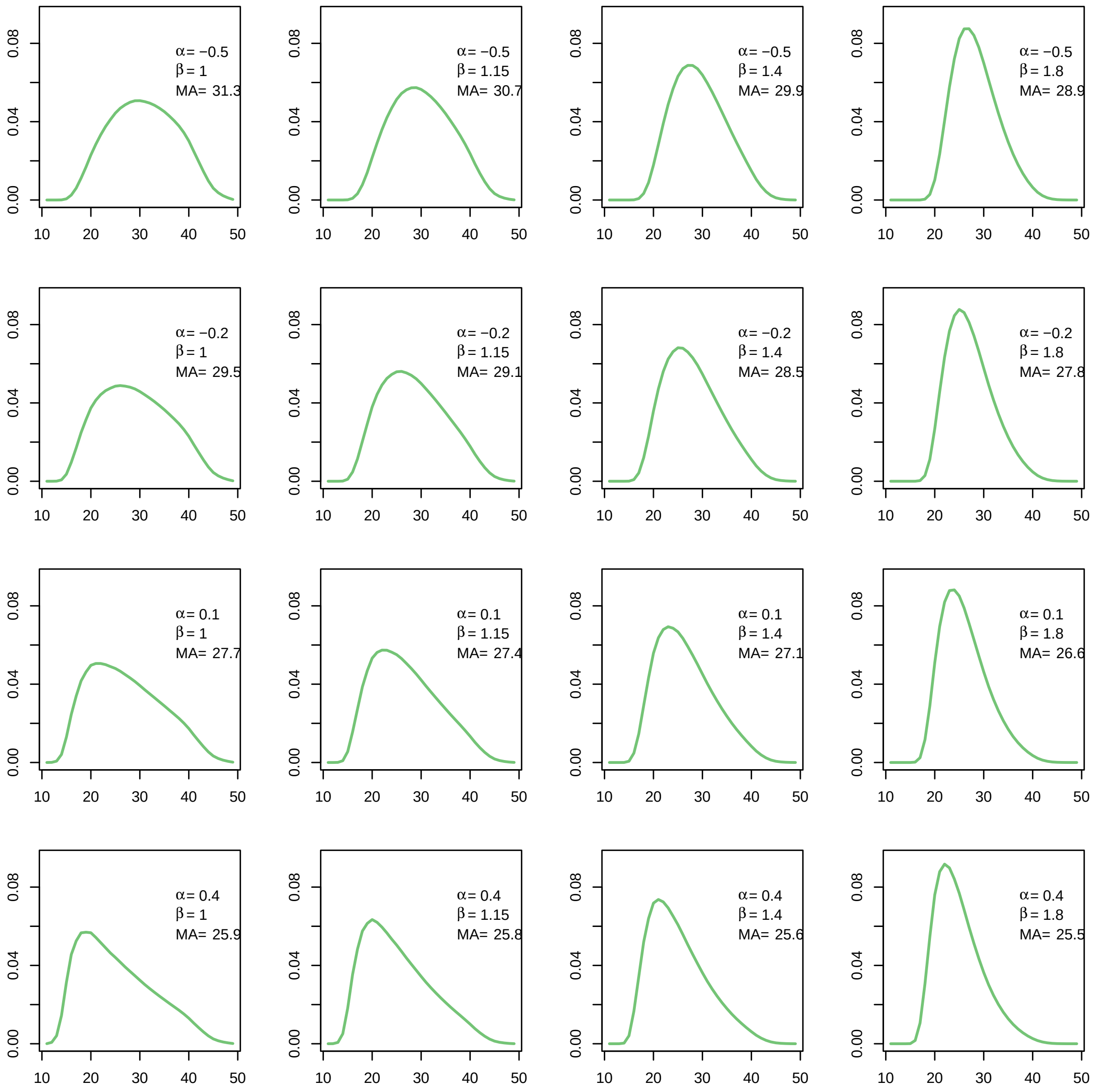

Figure A2 – *Age patterns of fertility introduced in the simulations, Booth standard [3] - MA is the mean age of the fertility schedule*

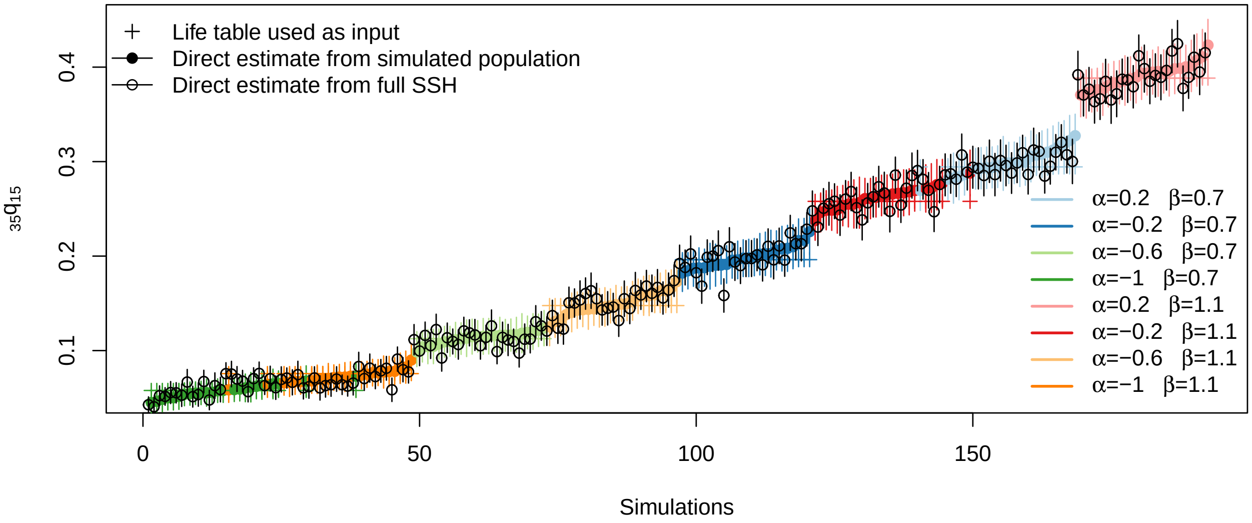

Figure A3 – *Probability of dying between ages 15 and 50 ($_{35}q_{15}$) introduced in the simulations, re-estimated from deaths in the overall populations and estimated directly from full SSH, for the different mortality age patterns*

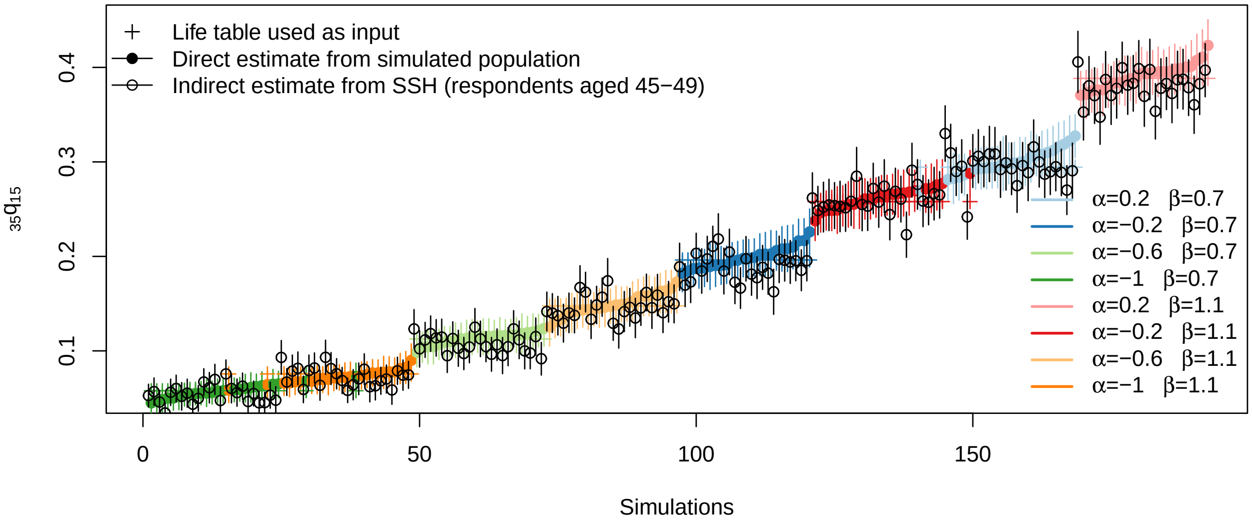

Figure A4 – *Probability of dying between ages 15 and 50 ($_{35}q_{15}$) introduced in the simulations, re-estimated from deaths in the overall populations, and estimated indirectly from adult lifetime proportions, for the different mortality age patterns*

References

[1] I. M. Timæus, B. Zaba, and M. Ali, "Estimation of adult mortality from data on adult siblings," in *Brass Tacks: Essays in Medical Demography* (B. Zaba and J. Blacker, eds.), pp. 43–66, London: Athlone Press, 2001.

[2] W. Brass, "On the scale of mortality," in *Biological Aspects of Demography* (W. Brass, ed.), pp. 69–110, London: Taylor et Francis, 1971.

[3] H. Booth, "Transforming Gompertz's function for fertility analysis: the development of a standard for the relational Gompertz function," *Population Studies*, vol. 38(3), pp. 495–506, 1984.

[4] J. W. Vaupel, "Biodemography of human ageing," *Nature*, vol. 464, pp. 536–542, Mar. 2010.
